# Large-scale randomized experiments reveals that machine learning-based instruction helps people memorize more effectively

**DOI:** 10.1038/s41539-021-00105-8

**Published:** 2021-09-06

**Authors:** Utkarsh Upadhyay, Graham Lancashire, Christoph Moser, Manuel Gomez-Rodriguez

**Affiliations:** 1 Max Planck Institute for Software Systems, Kaiserslautern, Germany; 2 Reasonal DE GmbH, Berlin, Germany; 3 Swift Management AG, Basel, Switzerland

**Keywords:** Long-term memory, Learning and memory, Forgetting

## Abstract

We perform a large-scale randomized controlled trial to evaluate the potential of machine learning-based instruction sequencing to improve memorization while allowing the learners the freedom to choose their review times. After controlling for the length and frequency of study, we find that learners for whom a machine learning algorithm determines which questions to include in their study sessions remember the content over ~69% longer. We also find that the sequencing algorithm has an effect on users’ engagement.

## Main text

The greater degree of personalization offered today by learning apps promises to facilitate the design and implementation of automated, data-driven teaching policies that adapt to each learner’s knowledge over time. However, to fulfill this promise, it is necessary to develop adaptive data-driven models of the learners, which accurately quantify their knowledge, and efficient methods to find teaching policies that are optimal under the learners’ models^1,2^.

In this context, research in the computer science literature has been typically focused on finding teaching policies that either enjoy optimality guarantees under simplified mathematical models of the learner’s knowledge^3–7^, adapt empirically to learners^8–10^, or optimize engagement^11,12^. In contrast, research in cognitive sciences has focused on measuring the effectiveness of a variety of heuristics to optimize the review times informed by psychologically valid models of the learner’s knowledge using (usually small) randomized control trials^13–17^. Only very recently, Tabibian et al.^18^ has introduced a machine learning modeling framework that bridges the gap between both lines of research—their framework can be used to determine the provably optimal review times under psychologically valid models of the learner’s memory state whose parameters are estimated from real review and recall data using a variant of half-life regression^12^. However, in the evaluation of their framework, the authors resort to a natural experiment using data from a popular language-learning online platform rather than a randomized control trial, the gold standard in the cognitive sciences literature. As a result, it has been argued that, in an interventional setting, an actual learner following the rate of study may fail to achieve optimal performance^1^.

We perform a large-scale randomized controlled trial involving ~50,700 learners of at least 18 years of age in Germany who use an app to study for the written portion of the driver’s permit from December 2019 to July 2020 and gave consent to participate in the trial. The goal of the randomized controlled trial is to evaluate to what extent a machine learning algorithm that builds upon Tabibian et al. can help people learn and remember more effectively. However, rather than optimizing the rate of study as in Tabibian et al., which is typically chosen by the learner, the algorithm determines which questions to include in a learner’s sessions of study over time. To facilitate research at the intersection of cognitive science and machine learning, we are releasing open-source implementation of our algorithm and all the data gathered during our randomized control trial.

During the randomized controlled trial, each learner was randomly assigned to a ‘select’, a ‘difficulty’, or a ‘random’ group throughout her entire usage of the app (Refer to Supplementary Information for more details on the random assignment). In the ‘select’ group (*n* = 10,151 learners), the questions of each study session were chosen using our machine learning algorithm. In the ‘difficulty` group (*n* = 34,029), they were chosen in circular order proportionally to the initial difficulty, i.e., easier questions first. In the ‘random` group (*n* = 13,600), they were chosen uniformly at random with replacement. The only difference in app functionality across groups was due to the item selection algorithm and learners do not know to which item selection algorithm they have been assigned. Moreover, in the ‘select’ group, as long as there were questions that the learner has not reviewed at least once, these were chosen first in order of initial difficulty, i.e., easier question first. By the end of the randomized controlled trial, we recorded more than ~16.75 million answers to ~1900 questions by ~50,700 learners in ~628,000 study sessions. Most of the learners were based in Germany (99.1%), they were evenly split between male (50.8%) and female (49.2%) and the most common age group was 18–24 (64.7%), followed by 25–34 (28%).

For consistency, we removed the data from the 6774 learners who reinstalled the app during the trial period and were assigned to a different group after the re-installation (or installed the app on different devices). Moreover, since we do not expect any algorithm to help learners who are cramming for tests, we do not use data from the 32,445 learners who used the app for less than 2 days. After these preprocessing steps, the resulting dataset contains ~894,000, ~3.3 million, and ~693,000 unique (learner, question) reviewing sequences due to 1564, 7582, and 2335 learners, respectively (refer to Supplementary Information for more details).

**Fig. 1:**
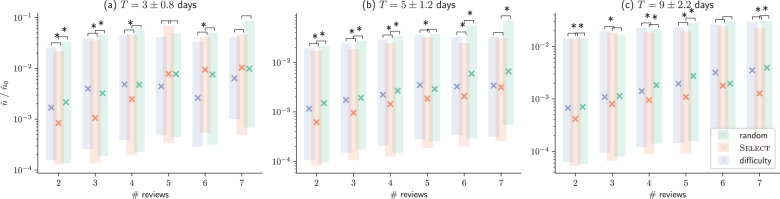
Normalized empirical forgetting rate. (Lower is better). Each triplet of bars in the figures corresponds to (learner, question) pairs in which the learner reviewed the question the same number of times (# reviews) for approximately the same period of time (T). Boxes indicate 25% and 75% quantiles and crosses indicate median values, where lower values indicate better performance. For each triple of bars, asterisk indicates a statistically significant difference (Matt–Whitney U-test, two-sided; *p* value = 0.05/36, Bonferroni correction).

We first compare learners of the ‘select’, ‘difficulty’ and ‘random’ groups in terms of normalized empirical forgetting rate^18^ (Fig. 1). After controlling for review time and number of reviews, the median normalized empirical forgetting rate for the learners in the ‘select’ group was lower than that of the learners in the ‘difficulty’ and ‘random’ groups in 83.5% of the cases and the decrease was statistically significant (Matt–Whitney U-test, two-sided; *p*-value = 0.05/36, Bonferroni correction) in 66.7% of the cases. Moreover, the median decrease in the median empirical forgetting rate for learners in the ‘select’ group was ~48% and ~40% when compared to learners in the ‘random’ and ‘difficulty’ groups, respectively, and the corresponding median increase in the median half-lives was ~92% and ~40%.

To further analyze the influence of each algorithm on the normalized empirical forgetting rate, we also fit the following regression model to the data for a fixed number of reviews:

1 $$\frac{\hat{n}}{\hat{{n}_{0}}}=c+{w}_{T}(T-{T}_{{{\mbox{median}}}})+{w}_{{{\mbox{difficulty}}}}{I}_{{{\mbox{difficulty}}}}+{w}_{{{\mbox{random}}}}{I}_{{{\mbox{random}}}},$$

where *c* is the intercept term, *T*_median_ is the median time for the last (test) review, *w*_*T*_ captures the impact of the spacing effect, and (*w*_random_, *w*_difficulty_) capture the effect of being assigned to ‘random’ or ‘difficulty’ group, respectively, relative to being assigned to the ‘select’ group. Table 1 summarizes the results, which suggest that the spacing effect holds true in aggregate, i.e., the coefficient associated with review time *T* − *T*_median_ is negative indicating that the more spaced the reviews are, the lower is the final normalized empirical forgetting rate. The results also suggest that, since *w*_difficulty_ > 0 and *w*_random_ > 0 across all #reviews, learners in the ‘select’ group have lower forgetting rate and, since the coefficients for the other groups seem to increase with number of reviews, the competitive advantage offered by our machine learning algorithm increases with the number of reviews.

**Table 1:** Multiple regression analysis to study the dependence the normalized empirical forgetting rate on the group assignment and the review time (Lower is better).

#reviews	*c*/10^−3^	*w*_*T*_/10^−3^	*w*_random_/10^−3^	*w*_difficulty_/10^−3^
2	2.5851	−0.0198	0.2434	0.2250
3	3.2134	−0.0220	0.1268	0.2731
4	3.0441	−0.0217	0.6490	0.6731
5	3.0312	−0.0220	1.1450	0.9979
6	3.6860	−0.0226	0.6395	0.7695
7	3.8292	−0.0251	1.1158	1.3460

We used Huber regression to determine the coefficients (see Supplementary Information for details).

In terms of engagement, learners of the ‘select’ (‘difficulty’) group were 50.6% (47.6%) more likely, in median, to return to the app within 4–7 days than learners of the ‘random’ group. However, learners of the ‘select’ group were also more likely to stop using the app in the initial 2 days than those of the other groups. Refer to Supplementary Information for more details.

While our results have direct implications for the learning of large sets of paired-associate items by young learners using machine learning-based instruction, we acknowledge that more research at the intersection of cognitive sciences and machine learning is needed to generalize our results to different populations of learners, different materials, or other tasks. In this context, it would also be interesting to compare our algorithm with stronger baselines and experiment with different feedback modalities to further understand which aspects are most responsible for the improved engagement and performance.

## Methods

### Modeling framework of spaced selection

Given a set of questions ${{{{{\mathcal{I}}}}}}$ whose answers a learner wants to learn, we represent each study session as a triplet $e:= (t,{{{{{\mathcal{S}}}}}},{r}_{{{{{{\mathcal{S}}}}}}})$, where ${{{{{\mathcal{S}}}}}}\subseteq {{{{{\mathcal{I}}}}}}$ is the set of questions that the learner reviewed at time *t* and ${r}_{{{{{{\mathcal{S}}}}}}}$ is a vector in which each entry corresponds to a question in the set ${{{{{\mathcal{S}}}}}}$ and indicates whether the learner recalled (*r* = 1) or forgot (*r* = 0) the answer to the question. Here, note that in the learning app that we used in our randomized experiment, the learner is tested in each study session, similar to most spaced repetition software and online platforms such as Mnemosyne, Synap, and Duolingo, and the seminal work of Roediger and Karpicke^19^.

Given the above representation, we keep track of the study times using a counting process *N*(*t*), which counts the number of study sessions up to time *t*. Following the literature on temporal point processes^20^, we characterize this counting process using its corresponding intensity *u*(*t*), i.e., *E*[*dN*(*t*)] = *u*(*t*)*dt*, and think of the set of questions ${{{{{\mathcal{S}}}}}}$ and vector ${r}_{{{{{{\mathcal{S}}}}}}}$ as its binary marks. Moreover, we utilize the well-known memory model from the psychology literature, the exponential forgetting curve model with binary recalls^21–24^, to estimate the probability *m*_*i*_(*t*) that a learner recalls (forgets) the answer to a question *i* at time *t*. Under the exponential forgetting curve model, the recall probability depends on the time since the last review Δ_*i*_(*t*) and the forgetting rate ${n}_{i}(t)\in {{\mathbb{R}}}^{+}$, which may depend on many factors, e.g., number of previous (un)successful recalls of the answer to the question. To estimate the value of the forgetting rate *n*_*i*_(*t*), we use a variant of half-life regression^12^ proposed by Tabibian et al.^18^ (refer to Supplementary Information).

The SELECT algorithm Given a set of questions ${{{{{\mathcal{I}}}}}}$, we cast the optimization of the study sessions as the search for the optimal selection probabilities ${p}_{i}(t):= {\mathbb{P}}[i\in {{{{{\mathcal{S}}}}}}]$ that minimize the expected value of a particular (quadratic) loss function *l*(***m***(*t*), ***n***(*t*), **Δ**(*t*), ***p***(*t*)) of the recall probability of the answers to the questions ${{{{{\boldsymbol{m}}}}}}(t)={[{m}_{i}(t)]}_{i\in {{{{{\mathcal{I}}}}}}}$, the forgetting rates ${{{{{\boldsymbol{n}}}}}}(t)={[{n}_{i}(t)]}_{i\in {{{{{\mathcal{I}}}}}}}$, the times since their last review ${{{{{\boldsymbol{\Delta }}}}}}(t)={[{{{\Delta }}}_{i}(t)]}_{i\in {{{{{\mathcal{I}}}}}}}$, and the selection probabilities ${{{{{\boldsymbol{p}}}}}}(t)={[{p}_{i}(t)]}_{i\in {{{{{\mathcal{I}}}}}}}$ over a time window (*t*_0_, *t*_*f*_].

To solve the above problem, we resort to the theory of stochastic optimal control of jumps and proceed similarly as in Tabibian et al.^18^. However, in contrast with Tabibian et al., rather than optimizing the rate of study, we optimize the selection probability of each question in each study session. In Supplementary Information, we show that, for each question $i\in {{{{{\mathcal{S}}}}}}$, the optimal selection probability is:

2 $${p}_{i}^{* }(t)=\frac{1}{\sqrt{q}}(1-{m}_{i}(t))$$

where *q* ≥ 1 is a given parameter, which trades off recall probability upon review and the size of the study sessions—the higher its value, the shorter the study sessions. In practice, in our randomized trial, the app presents questions according the order given by the selection probability and the user chooses the size of the study session. Therefore, our results are agnostic to the value of the parameter *q*.

Finally, since the optimal selection probability depends only on the recall probability, which is estimated using the exponential forgetting curve model, we can implement a very efficient procedure to construct study sessions, which we name SELECT (refer to Supplementary Information).

### Reporting summary

Further information on research design is available in the Nature Research Reporting Summary linked to this article.

## Supplementary information

Supplementary Information

Reporting Summary

## Data Availability

The data and code can be obtained at: https://github.com/Networks-Learning/spaced-selection.
