# Supplementary material for: Large-scale randomized experiments reveals that machine learning-based instruction helps people memorize more effectively
Source: NPJ Sci Learn. 2021 Sep 6;6:26. doi: 10.1038/s41539-021-00105-8 (PMC8421401; doi:10.1038/s41539-021-00105-8)
Supplement: Supplementary file 1 — Supplementary Information [file 41539_2021_105_MOESM1_ESM.pdf]

Large-scale randomized experiments reveals that machine learning-based instruction helps people memorize more effectively

Utkarsh Upadhyay[1,2], Graham Lancashire[3], Christoph Moser[3], and Manuel Gomez-Rodriguez[2]

Supplementary Information

Finding the optimal selection probabilities In this section, we derive the optimal selection probabilities $\mathbf{p}(t)$ for the power-law memory model [24] following a similar proof technique as in Tabibian et al. [18]. The derivation for the exponential memory model [23] can be done similarly.

Given a set of questions $\mathcal{I}$, we cast the optimization of the study sessions as the search for the optimal selection probabilities $p_i(t) := \mathbb{P}[i \in \mathcal{S}]$ for each question $i \in \mathcal{I}$ that minimize the expected value of a particular (convex) loss function $l(\boldsymbol{m}(t), \boldsymbol{n}(t), \boldsymbol{\Delta}(t), \boldsymbol{p}(t))$ of the recall probability of the answers to the questions $\boldsymbol{m}(t) = [m_i(t)]_{i\in\mathcal{I}}$, the forgetting rates $\boldsymbol{n}(t) = [n_i(t)]_{i\in\mathcal{I}}$, the times since their last review $\boldsymbol{\Delta}(t) = [\Delta_i(t)]_{i\in\mathcal{I}}$, and the selection probabilities $\boldsymbol{p}(t) = [p_i(t)]_{i\in\mathcal{I}}$ over a time window $(t_0, t_f]$, i.e.,

$$\text{minimize}_{p(t_0,t_f]} \quad \mathbb{E}\left[\phi(\boldsymbol{m}(t), \boldsymbol{n}(t), \Delta(t)) + \int_{t_0}^{t_f} l(\boldsymbol{m}(\tau), \boldsymbol{n}(\tau), \boldsymbol{\Delta}(\tau), \boldsymbol{p}(\tau))\, d\tau\right] \tag{3}$$

where $p(t_0, t_f]$ denotes the selection probabilities from t_0 to t_f, the expectation is taken over all possible realizations of the selection probabilities, the counting process $N(t)$ and the recalls of the answers to the questions, the loss function is nonincreasing (nondecreasing) with respect to the recall probabilities and the times since their last review (forgetting rates and selection probabilities) so that it rewards long-lasting learning while limiting the number of reviews, and $\phi(\boldsymbol{m}(t), \boldsymbol{n}(t), \Delta(t))$ is an arbitrary penalty function. Here, note that the rate of study session $u(t)$ is unknown and is not under our control, i.e., the learner chooses when to study.

To solve the above problem, we first express the dynamics of the forgetting rates $n_i(t)$ using the following stochastic differential equation (SDE) with jumps:

$$dn_i(t) = -\alpha_i n_i(t) r_i(t) dN_i(t) + \beta n_i(t)(1 - r_i(t)) dN_i(t) \tag{4}$$

where $E[dN_i(t)] = p_i(t)u(t)\, dt$. Now, we can use the above expression, the expression of the recall probability under the power-law forgetting curve model, i.e., $m_i(t) := \mathbb{P}[r_i(t) = 1] = (1 + \Delta_i(t))^{-n_i(t)}$, and Itö's calculus to express the dynamics of the recall probabilities $m_i(t)$ and the times since the last reviews $\Delta_i(t)$ using also SDEs with jumps, i.e.,

$$dm_i(t) = -\frac{n_i(t) m_i(t) dt}{(1 + \Delta_i(t))} + (1 - m_i(t)) dN_i(t) \tag{5}$$

$$d\Delta_i(t) = dt - \Delta_i(t) dN_i(t) \tag{6}$$

Then, given the above expressions, we can decompose the optimization problem defined by Eq. 3 into $|\mathcal{I}|$ independent problems, i.e.,

$$\underset{p_i(t_0,t_f]}{\text{minimize}} \ \mathbb{E}_{(N_i,r_i)(t_0,t_f]}\big[\phi(m_i(t_f), n_i(t_f), \Delta_i(t_f)) + \int_{t_0}^{t_f} \ell(m_i(\tau), n_i(\tau), \Delta_i(\tau), p_i(\tau))\, d\tau\big], \tag{7}$$

[1]Reasonal DE GmbH, Rosenthaler Str. 13, 10119 Berlin, Germany
[2]Max Planck Institute for Software Systems,Paul-Ehrlich-Strasse G26, D-67663 Kaiserslautern, Germany
[3]Swift Management AG, Feldbergstrasse 42, 4057 Basel, Switzerland

which can be solved separately.

Given a fixed question i, we denote $m(t) = m_i(t)$, $n(t) = n_i(t)$, $\Delta(t) = \Delta_i(t)$ and $p(t) = p_i(t)$, define the optimal cost-to-go function $J(m(t), n(t), \Delta(t), t)$ for the corresponding optimization problem as

$$J(m(t), n(t), \Delta(t), t) = \min_{p(t,t+dt]} \mathbb{E}[J(m(t+dt), n(t+dt), \tau(t+dt), t+dt)] + \ell(m(t), n(t), \Delta(t), p(t))dt \quad (8)$$

and use Bellman's principle of optimality to derive the corresponding HJB equation. In particular, we can first rewrite the above equation as

$$0 = \min_{p(t,t+dt]} \mathbb{E}[dJ(m(t), n(t), \Delta(t), t)] + \ell(m(t), n(t), \Delta(t), p(t))dt, \quad (9)$$

where $dJ(m(t), n(t), \Delta(t), t) = J(m(t+dt), n(t+dt), \Delta(t+dt), t+dt) - J(m(t), n(t), \Delta(t), t)$, and then use the following technical Lemma, which can be proved using Itö's calculus, to differentiate J with respect to their parameters.

Lemma 1. *Let $x(t)$, $y(t)$, $k(t)$ be three jump-diffusion processes defined by the following jump SDEs:*

$$\begin{aligned} dx(t) =& f(x(t), y(t), k(t), t)dt + g(x(t), y(t), k(t), t)z(t)dN(t) + h(x(t), y(t), k(t), t)(1 - z(t))dN(t) \\ dy(t) =& p(x(t), y(t), k(t), t)dt + q(x(t), y(t), k(t), t)dN(t) \\ dk(t) =& s(x(t), y(t), k(t), t)dt + v(x(t), y(t), k(t), t)dN(t) \end{aligned}$$

where $N(t)$ is a jump process and $z(t) \in \{0, 1\}$. If function $F(x(t), y(t), k(t), t)$ is once continuously differentiable in $x(t)$, $y(t)$, $k(t)$, and t, then,

$$\begin{aligned} dF(x(t), y(t), k(t), t) = & (F_t + fF_x + pF_y + sF_k)(x(t), y(t), k(t), t)dt \\ & + [F(x+g, y+q, k+v, t)z(t) + F(x+h, y+q, k+v, t)(1 - z(t)) - F(x, y, t)]\, dN(t), \end{aligned}$$

where for notational simplicity we dropped the arguments of the functions f, g, h, p and q.

More specifically, we can write $dJ(m(t), n(t), \Delta(t), t)$ in Eq. 9 as

$$\begin{aligned} dJ(m(t), n(t), \Delta(t), t) = & J_t(m(t), n(t), \Delta(t), t) - \frac{n(t)m(t)}{\Delta(t)+1} J_m(m(t), n(t), \Delta(t), t) + J_\tau(m, n, \tau, t) \\ & + [J(1, (1-\alpha)n(t), 0, t)r(t) + J(1, (1+\beta)n(t), 0, t)(1 - r(t)) - \\ & J(m(t), n(t), \Delta(t), t)]dN(t). \end{aligned}$$

and thus write the HJB equation as:

$$\begin{aligned} 0 = & J_t(m(t), n(t), \Delta(t), t) - \frac{n(t)m(t)}{\Delta(t)+1} J_m(m(t), n(t), \Delta(t), t) + J_\tau(m(t), n(t), \Delta(t), t) \\ & + \min_{u(t,t+dt]} \big\{ \ell(m(t), n(t), u(t)) \\ & + [J(1, (1-\alpha)n(t), 0, t)m(t) + J(1, (1+\beta)n(t), 0, t)(1 - m(t)) - J(m(t), n(t), \Delta(t), t)]\, p(t)u(t) \big\} \end{aligned} \quad (10)$$

To solve the above differential equation, we need to define the loss ℓ. Following the literature on stochastic control, we consider the following quadratic form, which penalizes quadratically the probability of unsuccessful recall of the answer to the question upon review and the probability of reviewing the question:

$$\ell(m(t), n(t), \Delta(t), p(t)) = \frac{1}{2}(1 - m(t))^2 u(t) + \frac{1}{2} q\, p^2(t) u(t) \quad (11)$$

where $q \geq 1$ is a given parameter, which trades off recall probability upon review and the size of the study sessions—the higher is its value, the shorter the study sessions.

Now, if we plug in the above loss into the HJB equation and set its derivative with respect to $p(t)$ to zero to derive the optimal $p^*(t)$, we obtain:

$$p^*(t) = \frac{1}{q}\left[J(m(t), n(t), \Delta(t), t) - J(1, (1-\alpha)n(t), 0, t)m(t) - J(1, (1+\beta)n(t), 0, t)(1-m(t))\right]_{(0,q)}, \quad (12)$$

where the operator $[\cdot]_{(a,b)} := \min(\max(a, \cdot), b)$ is required to ensure that $0 \leq p(t) \leq 1$. However, we will simplify these constraints to only positivity constraint and later verify that, by appropriately setting the tuning parameter q, we can make $p^*(t) \leq 1$ as well. Hence, if we plug in

$$p^*(t) = \frac{1}{q}\left[J(m(t), n(t), \Delta(t), t) - J(1, (1-\alpha)n(t), 0, t)m(t) - J(1, (1+\beta)n(t), 0, t)(1-m(t))\right]_+ . \quad (13)$$

back into the HJB equation, we find that the optimal cost-to-go J needs to satisfy the following nonlinear differential equation:

$$\begin{aligned} 0 &= J_t(m(t), n(t), \Delta(t), t) - \frac{n(t)m(t)}{\Delta(t)+1} J_m(m(t), n(t), t) + J_\tau(m(t), n(t), \Delta(t), t) + \frac{1}{2}(1-m(t))^2 \\ &- \frac{u(t)}{2q}\left[J(m(t), n(t), \Delta(t), t) - J(1, (1-\alpha)n(t), 0, t)m(t) - J(1, (1+\beta)n(t), 0, t)(1-m(t))\right]_+^2 . \end{aligned}$$

To solve it, we rely on the following technical Lemma:

Lemma 2. *Consider the following family of losses with parameter $d > 0$,*

$$\begin{aligned} \ell_d(m(t), n(t), p(t)) &= h_d(m(t), n(t), \Delta(t)) + g_d^2(m(t), n(t)) + \frac{1}{2} q\, p^2(t) u(t), \\ g_d(m(t), n(t)) &= \sqrt{\frac{u(t)}{2}}\left[c_2 \frac{\log(d)}{-m(t)^2 + 2m(t) - d} - c_2 \frac{\log(d)}{1-d} + c_1 m(t) \log\left(\frac{1+\beta}{1-\alpha}\right) - c_1 \log(1+\beta)\right], \\ h_d(m(t), n(t), \Delta(t)) &= -\sqrt{q} \frac{m(t)n(t)}{1+\Delta(t)} c_2 \frac{(-2m(t)+2)\log(d)}{(-m(t)^2 + 2m(t) - d)^2} \end{aligned} \quad (14)$$

where $c_1, c_2 \in \mathbb{R}$ are arbitrary constants. Then, the cost-to-go $J_d(m(t), n(t), \Delta(t), t)$ that satisfies the HJB equation, defined by Eq. 10, is given by:

$$J_d(m(t), n(t), \Delta(t), t) = \sqrt{q}\left(c_1 \log(n(t)) + c_2 \frac{\log(d)}{-m(t)^2 + 2m(t) - d}\right) \quad (15)$$

$$\Longrightarrow \frac{\partial J_d(m(t), n(t), \Delta(t), t)}{\partial t} = 0 \quad (16)$$

$$\frac{\partial J_d(m(t), n(t), \Delta(t), t)}{\partial \Delta} = 0 \quad (17)$$

$$\frac{\partial J_d(m(t), n(t), \Delta(t), t)}{\partial m(t)} = -c_2 \frac{(-2m(t)+2)\log(d)}{(-m(t)^2 + 2m(t) - d)^2} \quad (18)$$

and the optimal intensity is given by:

$$p^*(t) = q^{-1/2}\left[c_2 \frac{\log(d)}{-m(t)^2 + 2m(t) - d} - c_2 \frac{\log(d)}{1-d} + c_1 m(t) \log\left(\frac{1+\beta}{1-\alpha}\right) - c_1 \log(1+\beta)\right]_+ .$$

Proof. Consider the family of losses defined by Eq. 14 and the functional form for the cost-to-go defined by Eq. 15. Then, for any parameter value $d > 0$, the optimal intensity $p_d^*(t)$ is given by

$$\begin{aligned} p_d^*(t) &= \frac{1}{q}\left[J_d(m(t), n(t), \Delta(t), t) - J_d(1, (1-\alpha)n(t), 0, t)m(t) - J_d(1, (1+\beta)n(t), 0, t)(1-m(t))\right]_+ \\ &= \frac{1}{\sqrt{q}}\left[c_2 \frac{\log(d)}{-m^2 + 2m - d} - c_2 \frac{\log(d)}{1-d} + c_1 m(t) \log\left(\frac{1+\beta}{1-\alpha}\right) - c_1 \log(1+\beta)\right]_+ , \end{aligned}$$

Algorithm 1 SELECT Find the probability of selection of an item for study for one user

Input: Set of items $\mathcal{J}$, number of (un)successful recalls $n_i^{\checkmark}(t)$ and $n_i^{\times}(t)$, initial difficulties $\{n_i(0)\}$, last review times $\{t_i\}$ and parameters α, β and q.
Output: Probability of selection of each item $\mathbf{p}(t)$.
$\mathbf{p}(t) \leftarrow \mathbf{0}$
for $i \in \mathcal{J}$ **do**
  $n_i(t) \leftarrow n_i(0)(1-\alpha)^{n_i^{\checkmark}(t)}(1+\beta)^{n_i^{\times}(t)}$
  $m_i(t) \leftarrow \exp\left(-n_i(t)(t-t_i)\right)$ ▷ Using exponential memory model
  $p_i(t) = \frac{1}{\sqrt{q}}(1-m_i(t))$
end for
return $\mathbf{p}(t)$

and the HJB equation is satisfied:

$$
\begin{aligned}
&\frac{\partial J_d(m,n,\Delta,t)}{\partial t} - \frac{mn}{1+\Delta}\frac{\partial J_d(m,n,\Delta,t)}{\partial m} + \frac{\partial J_d(m,n,\Delta,t)}{\partial \Delta} + h_d(m,n,\Delta) + g_d^2(m,n) \\
&- \frac{u}{2q}\left[J_d(m,n,\Delta,t) - J_d(1,(1-\alpha)n,0,t)m - J_d(1,(1+\beta)n,0,t)(1-m)\right]_+^2 \\
&= \frac{mn}{1+\Delta}\underbrace{\sqrt{q}c_2\frac{(-2m+2)\log(d)}{(-m^2+2m-d)^2}}_{\frac{\partial J_d(m,n,\Delta,t)}{\partial m}} + h_d(m,n,\Delta) + g_d^2(m,n) \\
&\quad - \frac{u}{2}\left[c_1\log(n) + c_2\frac{\log(d)}{-m^2+2m-d} - m\left(c_1\log(n(1-\alpha)) + c_2\frac{\log(d)}{1-d}\right) - (1-m)\left(c_1\log(n(1+\beta)) + c_2\frac{\log(d)}{1-d}\right)\right]_+^2 \\
&= \frac{mn}{1+\Delta}\sqrt{q}c_2\frac{(-2m+2)\log(d)}{(-m^2+2m-d)^2}\underbrace{-\sqrt{q}\frac{mn}{1+\Delta}c_2\frac{(-2m+2)\log(d)}{(-m^2+2m-d)^2}}_{h_d(m,n,\Delta)} \\
&\quad - \frac{u}{2}\left[c_2\frac{\log(d)}{-m^2+2m-d} - c_2\frac{\log(d)}{1-d} + c_1 m\log\left(\frac{1+\beta}{1-\alpha}\right) - c_1\log(1+\beta)\right]_+^2 \\
&\quad \underbrace{+\frac{u}{2}\left[c_2\frac{\log(d)}{-m^2+2m-d} - c_2\frac{\log(d)}{1-d} + c_1 m\log\left(\frac{1+\beta}{1-\alpha}\right) - c_1\log(1+\beta)\right]_+^2}_{g_d(m,n)^2} = 0,
\end{aligned}
$$

where for notational simplicity $m = m(t)$, $n = n(t)$, $\Delta = \Delta(t)$ and $u = u(t)$. □

More specifically, note that $\lim_{d\to 1} l_d(m(t),n(t),p(t)) = \frac{1}{2}(1-m(t))^2 u(t) + \frac{1}{2}q\,p^2(t)u(t)$ and thus

$$
p^*(t) = \lim_{d\to 1} p_d^*(t) = \frac{1}{\sqrt{q}}\left[c_1 m(t)\log\frac{1+\beta}{1-\alpha} - c_1\log(1+\beta) - c_2)\right]
$$

Then, if we set $c_1 = \frac{1}{\log\frac{1-\alpha}{1+\beta}}$ and $c_2 = \frac{\log(1-\alpha)}{\log\frac{1-\alpha}{1+\beta}}$, we can readily conclude that the optimal selection probability is given by:

$$
p^*(t) = \frac{1}{\sqrt{q}}(1-m(t)) \tag{19}
$$

Finally, note that $1-m(t) \in [0,1]$ and hence, as long as $q \geq 1$, we have $p^*(t) \leq 1$, which will satisfy the constraints required in Eq. 12.

Learners' assignments to groups The assignment to the various groups was done by Firebase Optimizer and was not done uniformly at random. Whenever new learners arrive, they get assigned to a group at random, however, the assignment probabilities are chosen by the Firebase Optimizer and may change over time. The assignment probabilities do not depend on any feature of the learner being assigned.

We do not have data to access to which extent participants studied materials outside the app. However, we do not have any reason to believe that learners in one group or another study materials outside more than others.

Parameter estimation of memory models We utilize two well-known memory models from the psychology literature, the exponential and the power-law forgetting curve models [14, 15], to estimate the probability $m_i(t)$ that a learner recalls (forgets) the answer to a question i at time t. Under both models, the recall probability $m_i(t)$ depends on the time since the last review $\Delta_i(t)$ and the forgetting rate $n_i(t) \in \mathbb{R}^+$, which may depend on many factors, e.g., number of previous (un)successful recalls of the answer to the question. To estimate the value of the forgetting rate $n_i(t)$, we use (a variant of) half-life regression [11].

Half-life regression implicitly assumes that: (i) each question has an initial forgetting rate $n_i(0)$, which captures the difficulty of the question; (ii) a successful recall of the answer to a question i at time t' during a review change the forgetting rate by $(1-\alpha_i)$, i.e., $n_i(t) = (1-\alpha_i)\, n_i(t')$, $0 \leq \alpha_i \leq 1$; and, (iii) an unsuccessful recall changes the forgetting rate by $(1+\beta_i)$, i.e., $n_i(t) = (1+\beta_i)\, n_i(t')$, $\beta_i \geq 0$.

In our randomized control trial, we fit a single set of parameters α and β for all questions and a different initial forgetting rate $n_i(0)$ per question using the study sessions of all the learners who used the app from February 2019 to June 2019. To this end, we use the variant of half-life regression proposed by Tabibian et al. [See Appendix, Section 8] and perform a grid search to determine the optimal values of the hyper-parameters.

In terms of mean average error (MAE) in predicting the recall of items and in terms of correlation between the predicted and empirically observed half-life for the items (COR_h), the exponential model clearly outperforms the power-law model (MAE 0.139 vs 0.282 and COR_h 0.611 vs 0.571). However, in terms of Area Under the Curve (AUC), the power-law model performs slightly better than the exponential model (0.887 vs 0.901). Given these results, we decided to use the exponential memory model to estimate the recall probability during our randomized controlled trial.

Quality metric: normalized empirical forgetting rate For each (learner, question) reviewing sequence, we compare learners from each of the groups using the empirical forgetting rate [12], defined as

$$\hat{n} = -\frac{\log \hat{m}(t_n)}{t_n - t_{n-1}},$$

where $t_n - t_{n-1}$ is the last retention interval and $\hat{m}(t) = \max(\epsilon, \min(1-\epsilon, r(t)))$, with $\epsilon = 0.01$, indicates whether the learner was able to or unable to remember the answer at time t (the results presented are agnostic to the exact constant ϵ chosen).

For a fairer comparison across questions, we normalize each empirical forgetting rate using the average empirical initial forgetting rate of the corresponding question at the beginning of the observation window across users who studied the question. We fit one base forgetting rate per item, rather than per subject, to avoid a cold start problem (*i.e.*, when a new learner arrives to the platform, there is no subject data about the learner). Hence, our model does not take into account inter-subject variance in learning abilities. It should be noted that if enough individual subject data would be available, it might be possible to increase the accuracy of the memory model we used by considering subject specific parameters. However, such a memory model would be of little use with new learners.

Long term users of the app When we remove the users who used the app for fewer than 2 days from our dataset, the number of users in the 'select' group went from 7,030 to 1,564, in 'difficulty' group went from 27,983 to 7,582, and in 'random' group went from 8,858 to 2,335. This indicates a reduction of about 78% for 'select', 73% for 'difficulty', and 74% for 'random'. This discrepancy in the relative reduction can partially be explained by a corresponding slight decrease in the number of crash-free users every day, i.e., a

median decrease of 0.17% per day between 'difficulty' and 'select'. The marginally higher complexity of the 'select' algorithm initially caused over/under flow problems which could have prompted learners to stop using the app before they reached the 2 days of learning activity. Nevertheless, in terms of engagement (*retention* in Firebase Analytics), learners of the 'select' ('difficulty') group were 50.6% (47.6%) more likely, in median, to return to the app within 4–7 days than learners of the 'random' group. Moreover, Firebase Analytics estimated that, with probability 80%, our algorithm was the top performer in terms of engagement. This seems to suggests that the initial engagement of learners assigned to the 'select' group dropped, but if the learners persisted, then those assigned to the 'select' group were more likely to continue using the app in the next $4-7$ days.

Regression analysis of results In the results presented in the main section, we controlled for the group of the learners, the number of reviews, and time spent reviewing by putting (learners, items) in separate bins. In this section, we present another way to control for the same. We perform multiple regression to analyze the dependence of the normalized empirical forgetting rate on the time spent reviewing (modelled as continuous variable) and the group learners were assigned to (modelled as indicator variables). This analysis is not perfect as it is known that the normalized empirical forgetting rate is not linearly related to the variables, but the analysis nevertheless leads to interesting insights.

We perform different regressions for different number of reviews. This can be interpreted as including an interaction term with the total number of reviews in the regression instead of treating it as a categorical or ordinal variable. For a fixed number of reviews N, we fit the following regression model to the data

$$\frac{\hat{n}}{\hat{n}_0} = c + w_T(T - T_{\text{median}}) + w_{\text{difficulty}} I_{\text{difficulty}} + w_{\text{random}} I_{\text{random}} \tag{20}$$

where c is the intercept term, T_{median} is the median time for the last (test) review, w_T captures the impact of the spacing effect, and $(w_{\text{random}}, w_{\text{difficulty}})$ capture the effect of being assigned to 'random' or 'difficulty' group, respectively, relative to being assigned to the 'select' group. The intercept term c can be interpretted as the normalized forgetting rate for the 'select' group at the median time of the test review. We used the Huber loss ($\varepsilon = 1.35$, $\alpha = 0.001$) instead of the L2 loss to further reduce the effect of extreme outliers in the dataset.

Additional details on iTheorie Führerschein Auto Learners use the iTheorie Führerschein Auto to prepare for the written section of the driving lessons. When learners install the app, they are assigned to one of the three item selection algorithms randomly via the Firebase Optimizer. The learners do not know which item selection algorithm has been used to create their study sessions.

Upon starting the app, the learners are greeted with a screen where they can select the lessons they would like to take, shown in Supplementary Figure 1a. Once they select a category, a study session starts, and they are given questions to answer, as shown in Supplementary Figure 1b. The selection of items in each section is done using the algorithm assigned to the learner. A study session continues until the learner takes a break of longer than 5 minutes. Supplementary Figures 1c and 1d show the notification shown to the learner after a correct and incorrect answer respectively.

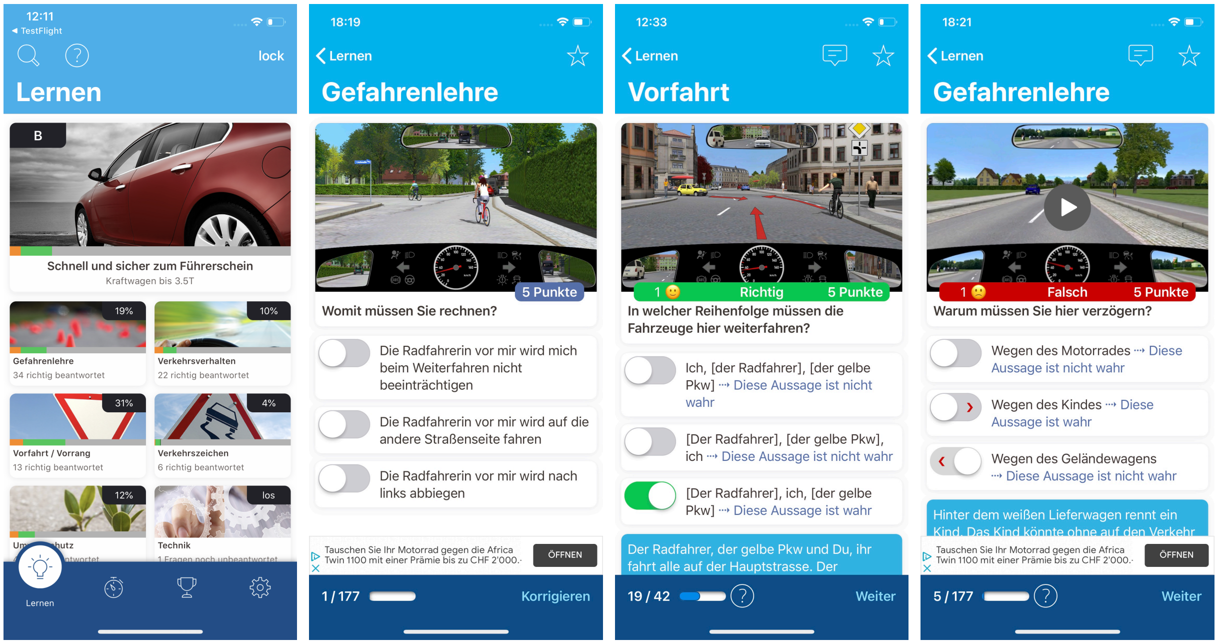

(a) Lesson selection (b) Session start (c) Correct response (d) Incorrect response

Supplementary Figure 1: The iTheory learning app.
